# The role of mesenchymal stem cells in attenuating inflammatory bowel disease through ubiquitination

**DOI:** 10.3389/fimmu.2024.1423069

**Published:** 2024-08-09

**Authors:** Hong Xi Liao, Xiaojun Mao, Lan Wang, Naijian Wang, Dickson Kofi Wiredu Ocansey, Bo Wang, Fei Mao

**Affiliations:** ^1^ Key Laboratory of Medical Science and Laboratory Medicine of Jiangsu Province, School of Medicine, Jiangsu University, Zhenjiang, Jiangsu, China; ^2^ Department of Laboratory Medicine, Lianyungang Clinical College, Jiangsu University, Lianyungang, Jiangsu, China; ^3^ The People’s Hospital of Danyang, Affiliated Danyang Hospital of Nantong University, Zhenjiang, Jiangsu, China; ^4^ Department of Laboratory Medicine, Danyang Blood Station, Zhenjiang, Jiangsu, China

**Keywords:** mesenchymal stem cell, exosome, inflammatory bowel disease, post-translational modification, ubiquitination

## Abstract

Inflammatory bowel disease (IBD), a condition of the digestive tract and one of the autoimmune diseases, is becoming a disease of significant global public health concern and substantial clinical burden. Various signaling pathways have been documented to modulate IBD, but the exact activation and regulatory mechanisms have not been fully clarified; thus, a need for constant exploration of the molecules and pathways that play key roles in the development of IBD. In recent years, several protein post-translational modification pathways, such as ubiquitination, phosphorylation, methylation, acetylation, and glycolysis, have been implicated in IBD. An aberrant ubiquitination in IBD is often associated with dysregulated immune responses and inflammation. Mesenchymal stem cells (MSCs) play a crucial role in regulating ubiquitination modifications through the ubiquitin-proteasome system, a cellular machinery responsible for protein degradation. Specifically, MSCs have been shown to influence the ubiquitination of key signaling molecules involved in inflammatory pathways. This paper reviews the recent research progress in MSC-regulated ubiquitination in IBD, highlighting their therapeutic potential in treating IBD and offering a promising avenue for developing targeted interventions to modulate the immune system and alleviate inflammatory conditions.

## 1 Introduction

Over the past 20 years, the incidence and prevalence of Inflammatory bowel disease (IBD) have increased in the newly industrialized countries of Asia, South America, the Middle East, and Africa, and the rate is particularly significant in South America and East Asia. Thus, IBD is gradually expanding to become a global disease of public health concern^1^. According to China’s epidemiological data, the incidence of IBD, ulcerative colitis (UC) and Crohn’s disease (CD), in the north of China is lower than in the south. For instance, the age-standardized incidence of IBD in Daqing City (Heilongjiang Province), Wuhan, and Guangzhou are 1.77/100,000, 1.96/100,000, and 3.14/100,000 respectively (1, 2). According to a study (3), the number of IBD patients in China will reach 1.5 million by 2025. Similar observations have been reported, with an estimated 6·8 million cases of IBD globally, where the USA had the highest age-standardized prevalence rate (464·5 [438·6–490·9] per 100 000 population), followed by the UK (449·6 [420·6–481·6] per 100 000) (4).

IBD has evolved from a common and fatal condition to a manageable chronic condition (5), which is divided into two broad categories, mainly UC and CD (6, 7). The main clinical manifestations are persistent abdominal pain, diarrhea, and bloody stool, accompanied by malnutrition, weight loss, mental distress, and other symptoms. If the course of the disease is prolonged, there is a possibility of colorectal cancer, a severe threat to human health. It has been observed that people with a history of digestive disease, a family history of IBD, and a functional imbalance in the body’s immune system are more likely to develop IBD than the general population (8). As a chronic non-specific intestinal inflammatory disease, IBD is associated with environmental, gut microbial, genetic, and immune factors. If IBD is not correctly diagnosed and treated in time, the disease becomes severe and lesions accumulate in organs of the body, with complications for disease such as colon cancer, coronary heart disease, primary sclerosing cholangitis, and phlebitis.

Ubiquitination is an important post-translational modification of proteins, and the enzymes involved mainly include the E1 ubiquitin-activating enzyme (E1), E2 ubiquitin binding enzyme (E2), and E3 ubiquitin-ligase (E3). Ubiquitin modification can regulate the localization and function of proteins in cells, degrade proteins, and regulate life activities such as signal transmission, gene expression regulation, cell proliferation, differentiation, apoptosis, inflammation and immunity (9). Abnormal ubiquitination can cause cancer, metabolic syndrome, neurodegenerative diseases, autoimmune diseases, inflammatory diseases, infections, and muscular dystrophy. Recent studies have reported associations between ubiquitination and/or deubiquitination and the onset and development of IBD (10). A genome-wide association analysis (GWAS) study showed that rare variants of E3 ligase RNF186 were associated with IBD (11). At the same time, the expression of ubiquitin mRNA in the colonic tissue of experimental colitis rats was significantly higher than that in the normal group. Therefore, abnormal ubiquitination may be one of the important mechanisms of colonic inflammation and immune damage in IBD.

In recent years, extracellular vesicles (Evs), as a strategy of “cell-free therapy,” have become a “new favorite” in research. With the development of various related technologies, more and more researchers have turned their attention to the use of Evs for disease diagnosis, prognosis, and therapeutic clinical applications. Mesenchymal stem cells (MSCs) and their derived exosomes (MSC-Exs) have been shown to play a significant role in the repair of various diseases, including IBD (12). MSCs and MSC-Exs play an important role in the information transmission process between damaged intestinal cells and can be involved in the regulation of intestinal inflammation and damage repair, showing great potential in the treatment of IBD (13). The main mechanism by which mesenchymal stem cells-derived extracellular vesicles (MSC-Evs) inhibit the activation of colon macrophages depends on the inhibition of NF-κB and iNOS transduction signals. After injection of MSC-Evs, the expression of NF-κB p65 in colon macrophages can be downregulated, and the production of NO, IL-1β, and IL-18 can be reduced, thus alleviating the symptoms of colitis (14, 15). At the same time, ubiquitination plays an essential role in the regulation of multiple biological functions, including inflammation. Studies in mice with colitis found that ubiquitin protein from inflammatory tissue is upregulated, and ubiquitin (Ub) is involved in the signaling pathway that regulates the expression of inflammatory factors (mTOR signaling pathway) (16). Wu et al. found that human umbilical cord mesenchymal stem cells-derived exosome (hucMSC-Ex) can down-regulate the expression level of ubiquitin protein. This, in turn, reduces NF-KB and mTOR activation (17). In addition, hucMSC-Ex can modulate the expression of polyubiquitination, including K48. It is concluded that MSC-Ex may play an anti-inflammatory role by regulating the level of ubiquitin modification. Currently, the product development and clinical translational application of MSCs and MSC-Exs are a hot topic in drug development, and cell-free therapy plays a unique role as a breakthrough clinical therapy technology. However, no literature currently summarizes the research progress of MSCs regulating ubiquitin modification to repair IBD. This review can provide a theoretical basis for Cell-free therapy to treat IBD through ubiquitin modification, which has important research value.

## 2 Mesenchymal stem cells

MSCs are a class of pluripotent stem cells with the function of self-renewal, self-proliferation, and multi-differentiation (18, 19). They positively express CD73, CD90, and CD105 and negatively express CD19, and CD45 (20). MSCs are derived from a wide range of sources, including bone marrow, adipose tissue, endometrial polyps, umbilical cord, amniotic fluid, and placenta (21). They generally possess the functions of inducing regeneration, maintaining general tissue homeostasis, and homing at target sites, which are their inherent characteristics (22). However, the differentiation and proliferation potential of MSCs from different sources may be very different (23).

MSCs can interact with cells of both the innate and adaptive immune systems. Evidence has shown that MSCs exert immunomodulatory functions by regulating the activation, proliferation, and differentiation of immune effector cells, including natural killer cells (NK), macrophages (Mø), dendritic cells (DC), B lymphocytes, and T lymphocytes (24). MSCs can down-regulate NKp30 and natural-killer group 2, member D (NKG2D), to inhibit the cytotoxic activity of resting NK cells, and the latter is an activated receptor involved in NK cell activation and target cell killing (25). MSCs can regulate Th1/Th2 balance (T helper cells) by influencing the levels of interleukin-4 (IL4) and interferon (IFN-γ) in effector T cells (26). MSCs can also reduce inflammation, improve tissue damage, and prevent infection by secreting a variety of immunomodulatory factors, including IFN-γ, prostaglandin E2 (PGE2), and growth factors (TGF-β, VEGF) (27). In conclusion, MSCs have strong immunomodulatory properties but are less immunogenic.

At present, MSCs are widely used in the research and treatment of various human diseases such as cardiovascular diseases, osteoarthritis, metabolic disease, etc. (28)). LAI et al. believe that MSCs play a role through their secreted products (29). It has been reported that MSCs can secrete a variety of Evs, including exosomes (30). In recent years, exosomes, as a promising substitute for MSCs, have attracted more attention, and have great research significance for experimental and clinical applications.

## 3 Biogenesis, composition, and characteristics of MSC-derived exosomes

Extracellular vesicles (Evs) secreted by cells are divided into apoptotic bodies, microvesicles, and exosomes based on their size, content, and formation mechanism (31). **Table 1** presents the classification and function of Evs. Evs are released by almost all living cells and are found in blood, urine, and bronchoalveolar lavage fluid (32). At present, exosomal research is the most attractive and constantly expanding. In 1983, the research team of Rose M. Johnstone (33), a professor in the Department of Biochemistry of McGill University in Canada, first found exosomes in sheep reticulocytes, which were considered to be cell follicles that could transport nonessential proteins between cells and were considered the “garbage” of cell metabolism. With further study, Johnstone named these small vesicles as exosomes in 1987.

**Table 1:** Classification and function of Evs.

	Exosomes	Microvesicles	Apoptotic Bodies
**Origin**	Endocytic pathway	Cell plasma membrane	Cell plasma membrane
**Size**	50–200 nm	100–1000 nm	1000–5000 nm
**Content of EVs**	Proteins and nucleic acids (mRNA, miRNA, IncRNA, and DNA fragments), lipids	Membrane proteins, phospholipids, RNA and other biomolecules	Cell membranes, DNA, coding and non-coding RNA, lipids, and containing specific vesicular membrane proteins,
**Functions**	Intercellular communication, affects the physiological and pathological state of host cells.	Cell recognition and signaling functions that help host cells transmit information and regulate immune responses	Phagocytosis, affects the cellular immune state and pathological process.

Exosomes, uniform in size and 50 to 200nm in diameter, have no cellular structure and are highly stable (34–36). Exosomes facilitate intercellular communication by carrying bioactive substances including mRNA, miRNA, IncRNA, DNA fragments, proteins, and lipids from parent cells, thereby regulating the activities of target cells. They positively express markers such as the tetraspanins protein family (CD63, CD81, and CD9), MVB biogenic proteins (Alix, TSG101, and ESCRT Complex), membrane transporters and heat shock proteins (HSP70, HSP90), lipid-associated proteins, etc. (37).

**Figure 1:**
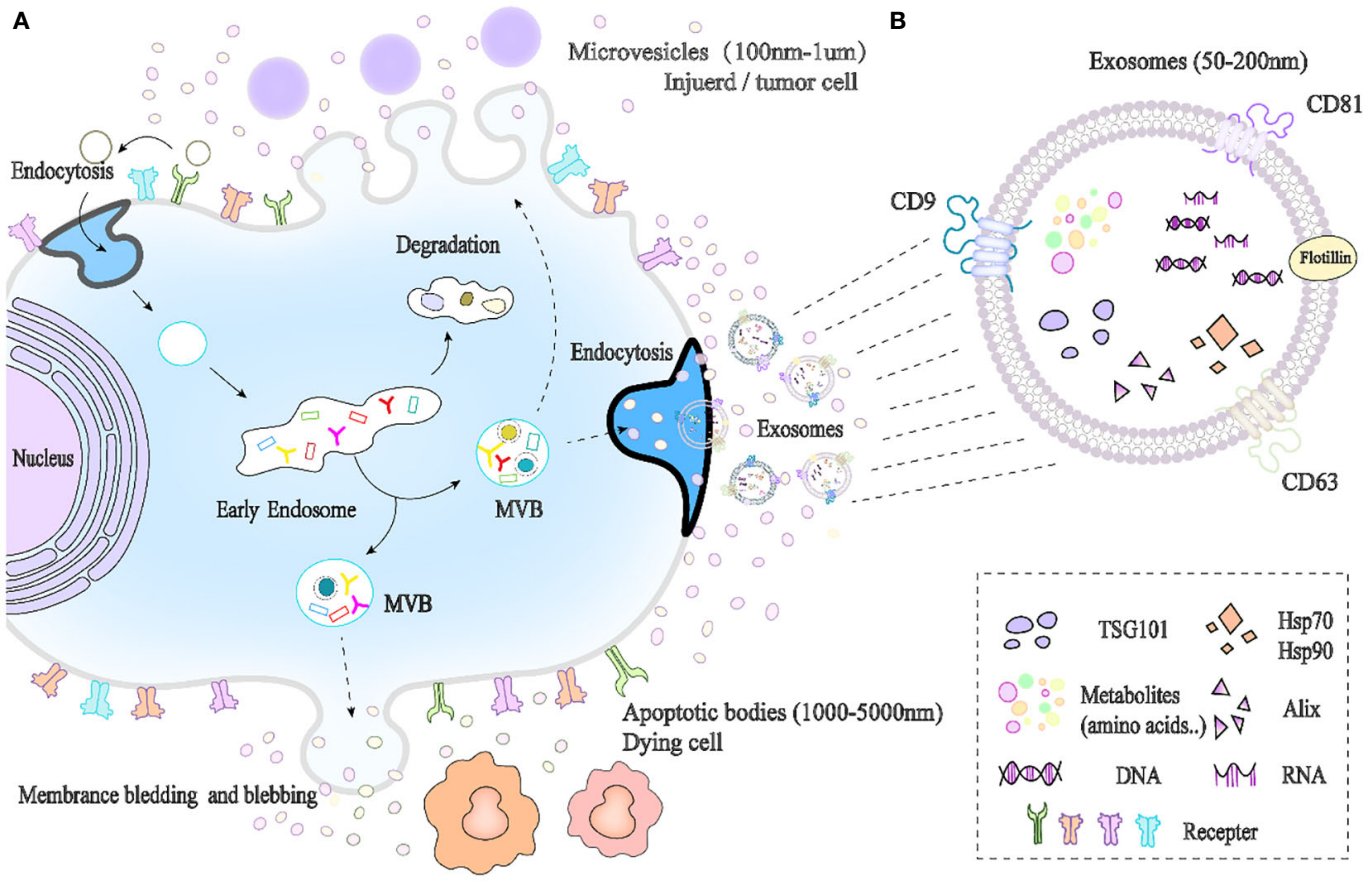
Biogenesis and composition of exosomes. **(A)** Exosomes originate from endosomal pathways, and extracellular substances enter cells through membrane invagination and endocytosis and then develop into early endosomes (ESEs), late endosomes (LSEs), and intracavitary vesicles (ILVs). Multiple intracavitary vesicles (ILVs) aggregate to form MVBs. MVBs can fuse with lysosomes to degrade and release content into the cytoplasm or be released outside the cell by budding through the cell membrane, and the latter is called exosomes. **(B)** Exosome composition.

Exosome biogenesis includes three main stages of endosomes, multivesicular body (MVBs) formation and exosome release (**Figure 1**), which involve double invagination of the plasma membrane (38, 39). Exosomes originate in the endosomal system of cells. Extracellular substances first enter the cell through membrane invagination and endocytosis, fuse with early endosomes (ESEs), and gradually develop and mature into late endosomes (LSEs). Late endosomal invagination leads to the emergence of intracavitary vesicles (ILVs), and multiple ILVs aggregate to form MVBs. MVBs are fused with the cell membrane and released outwards as exosomes in lipid bilayers.

Because exosomes can be detected in body fluids, they are considered noninvasive or minimally invasive biomarkers for disease diagnosis. Studies have implicated exosomes in the pathophysiology of several diseases (40). For example, Gui et al. found that compared with healthy controls, the expressions of miR-1 and miR19b-3p in CSF exosomes of Parkinson’s disease (PD) patients were down-regulated, while miR-153, miR-409–3p, and miR-10a5p were up-regulated (41). The exosomal miRNAs significantly correlated with the severity of PD and may be an effective biomarker for evaluating disease development in clinical PD patients. Exosomes can also be used as diagnostic markers of cancer. Roccaro et al. showed that the expression of miRNA-15a in bone marrow MSC-exosomes (BMMSC-Ex) of multiple myeloma patients is significantly down-regulated, which is closely related to the characteristics of multiple myeloma (42). In addition, the presence of high levels of Evs expressing TGF-β2 in the breast milk of normally lactating women can induce breast cancer. What is interesting is that exosomes can play a therapeutic role by delivering drugs themselves or as functional cargo for drug delivery (43, 44). For example, MSC-Exs are used in the treatment of graft-versus-host disease (GVHD), with significant therapeutic effects observed after repeated injection without serious side effects (45, 46).

MSC-Exs have similar biological functions to MSCs, playing an important role in improving tissue repair, immune regulation, inhibiting inflammatory response, and reducing apoptosis (47). They exhibit no tumorigenicity, and are easier to extract, modify, and store (48). MSC-Ex is a natural, non-toxic vesicle that can deliver mRNA, miRNA, and protein. Therefore, it has received extensive attention as a cell-free therapeutic carrier in treating autoimmune diseases, including IBD (49). MSC-Ex research has made substantial progress in the treatment of multiple sclerosis, type-1 diabetes, and other diseases (50–52) (**Table 2** shows the application of MSCs and MSC-Ex in various clinical diseases). In the DSS-induced IBD model, hucMSC-Exs treatment reduces the infiltration of macrophages in colon tissue and inhibits the expression of IL-7 (74). Moreover, hucMSC-Exs alleviate insulin secretion function in T2DM by reversing peripheral insulin resistance and alleviating β cell destruction, providing a new approach for T2DM treatment (60). In clinical treatment, MSC-Exs have obvious advantages over MSCs and may completely replace MSC therapy in the future.

**Table 2:** Applications of MSCs and MSC-Ex in various clinical diseases.

Disease	Study type	Treatment used	Observation	Reference
**Acute myocardial infarction**	Case Reports	MSCs	Improved	(53)
	Clinical Trial	MSCs	Improved	(54)
	Animal model	MSC-Ex	Improved	(55, 56)
**Ischemic heart disease**	Clinical Trial	MSCs	Improved	(57)
**Type 1 diabetes**	Animal model	Both	Improved	(52, 58)
	Clinical Trial	MSCs	Improved	(59)
**Type 2 diabetes**	Animal model	MSC-Ex	Improved	(60)
**Systemic lupus erythematosus**	Clinical Trial	MSCs	Improved	(61, 62)
**Graft versus** **host disease**	Animal model	MSCs	Improved	(63)
	Case Reports	MSCs	Improved	(47)
**Multiple Sclerosis**	Clinical Trial	MSCs	Improved	(64)
**Lung disease**	Clinical Trial	Both	Improved	(65–68)
**Alzheimer’s disease**	Animal model	MSC-Ex	Improved	(69, 70)
**Kidney injury**	Clinical Trial	MSCs	Improved	(71)
	Animal model	MSC-Ex	Improved	(72)
**Apoplexy**	Clinical Trial	MSCs	Improved	(73)
**IBD**	Animal model	MSC-Ex	Improved	(12, 74, 75)

## 4 Ubiquitination

Ubiquitination is an important process that regulates the normal expression of genes, along with phosphorylation, glycosylation, acetylation, amidation, etc. (76, 77) Ubiquitination modifies post-translated proteins (PTMs), including protein degradation, signal transduction, and DNA damage repair (78, 79). Ubiquitination modulates various cellular activities involved in inflammatory responses, innate or adaptive immune responses, and ribosomal functions, which are essential for many cell life processes (80).

Cellular processes depend on ubiquitin (Ub) and ubiquitin-like proteins (UBLs) in ubiquitination systems. Ub, a highly conserved small protein in eukaryotes, contains 76 amino acid residues and is a significant part of regulating the everyday life activities of biological proteins (81). The UBLs found so far include NEDD8 (neural-precursor-cell expressed developmentally down-regulated 8) and SUMO (1–5, small ubiquitin-like modifier). Although they have similar functions to Ub, the receptors and signaling molecules they contact are not the same and play different roles. Related studies have shown that NEDD8 which has the same homologous sequence (> 50%) as Ub, binds to Cullin, the subunit cullin of Cullin-ring ligase (the largest multi-unit E3s ubiquitase family, CRLs). After overactivation, it promotes the degradation of tumor suppressor factors (p21, p27) and the occurrence and evolution of cancer (82). Yang W et al. experimentally demonstrated that increased SUMOylating has a neuroprotective effect and seems necessary for survival, at least under certain conditions (ischemia) (83). This theory was further validated in animal models, which found that cerebral ischemia in mice leads to a significant increase in SUMO2/3 conjugates in the hippocampus and cerebral cortex, and a neuroblastoma cell model undergoing hypoxia/glucose deprivation followed by a short period of reoxygenation under the same conditions also exhibits significant increases in SUMO2/3 conjugation (84, 85).

From a more microscopic point of view, the Ub molecule itself contains seven lysine (Lys) residues, and the amino terminus of the Lys residues (K6, K11, K27, K29, K33, K48, and K63) on the substrate protein monopeptide can be labeled for monoubiquitination. **Table 3** summary of the different types of ubiquitination and their functions. When the Ub molecule forms a specific isopeptide bond with the carboxyl terminus of another Ub molecule through Lys residues, it is further coupled to form a multiubiquitin chain. Ubiquitin can also bind to non-lysine residues, such as cysteine (Cys). Ubiquitin chains with different links have different cellular functions, constituting the “ubiquitin code” of diversity and complexity (86). Monoubiquitination is generally associated with receptor internalization, while polyubiquitination is usually associated with proteasome degradation signaling (87). Related studies have found that K48 and K11 connected ubiquitin chains mediate substrate proteins to be digested into amino acids by soluble peptidase (26S proteasome complex) in cytoplasm or nucleus through a series of enzymatic reactions (88, 89). Many experimental studies have shown that the polyubiquitin chain of K63 is associated with non-proteasome functions, such as DNA repair, protein sorting, immune modulation, and regulation of the activation of the NF-κB signaling pathway, and also protects target proteins from multiple signaling pathway functions, including T cell receptors, Toll-like receptors (TLRs) and RIG-I-like receptor-mediated signal transduction (90–93). The mechanisms determining whether a protein is monoubiquitinated or polyubiquitinated are not fully understood and require further studies.

**Table 3:** Summary of the different types of ubiquitination and their functions.

	Ubiquitin type	Function
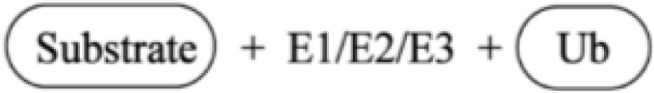	K11	Protein degradation, regulation of cell cycle, DNA damage, and signal transduction.
	K48	Protein degradation.
	K63	DNA repair, translation, signal transduction, and trafficking.
	Others	Protein degradation and DNA repair.

**Figure 2:**
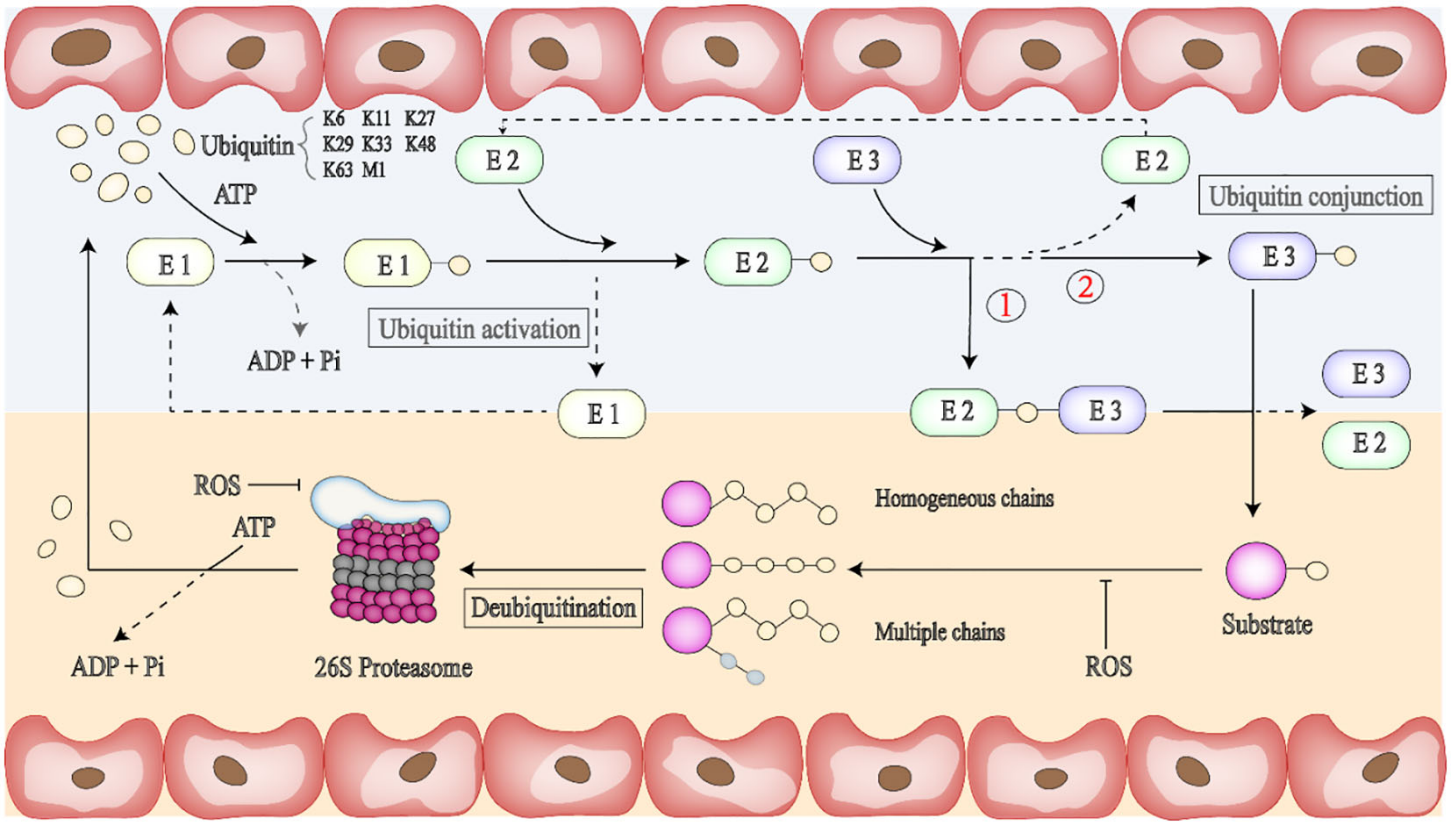
Binding of ubiquitin to protein substrates is a multi-step process. Ubiquitin molecules can be attached to ubiquitin or proteases containing lysine residues such as K6, K11, K27, K29, K33, K48, and K63. First, ATP provides energy, and the E1 enzyme activates ubiquitin and initiates the ubiquitination process. E2 enzyme can provide ubiquitin directly to the protein substrate through lysine (K) residues in the target protein. The third step is to bind the E2 ubiquitase-linked ubiquitin to the E3 ligase-linked target protein, and there are two binding forms, respectively,① E2-Ub-substrate protein-E3 or ② Ub substrate protein-E3. Ubiquitin protein ligase (E3) can promote the interaction with substrate proteins. Ubiquitination is a reversible post-translational protein modification; thus, the substrate carrying the ubiquitin chain can be deactivated by the UPS (Ubiquitin-proteasome system) or DUBs (Deubiquitinating enzymes), and free ubiquitin monomers can be re-recruited for the next round of ubiquitination. The ubiquitin chain usually determines the fate of ubiquitinated proteins.

Ubiquitination of proteins is accomplished through a series of continuous enzymatic reactions (94–96) (**Table 4**). For the enzymes required for ubiquitination, it is currently estimated that eukaryotic organisms have two E1 enzymes (UBA1 and UBA6), with approximately 30–50 E2 enzymes and more than 600 E3 enzymes. In a tertiary E1-E2-E3 enzyme-linked reaction in mammals, any two members of the E1 family can label all E2 with Ub, and 40 known E2 enzymes can further transmit Ub to the E3s family. Auxiliary E4 enzymes have been explored (97); When the substrate protein signals, the E1 enzyme activates ubiquitin and starts the ubiquitination process, which requires ATP to provide energy. Then, the E3 ubiquitin ligase specifically recognizes the substrate protein and guides the E2 conjugation-carried Ub to covalently bind to the substrate protein (**Figure 2**) (98). In the ubiquitination system, these three different types of enzymes cooperate to complete the task of modifying proteins. The ubiquitin activator E1 binds to the tail of the ubiquitin molecule, and regulates the downstream of the ubiquitination reaction. Ubiquitin coupling E2 enzyme controls the length and connection type during the assembly of ubiquitin chains, and K48 and K63-mediated polyubiquitination regulates the inflammatory development of the NF-κB signaling pathway. The specificity of the ubiquitin chain is widely believed to be determined by E2-E3 (RING-E3s) matching or substrate-E3 (HECT and with E6-APC) complexes (99).

**Table 4:** Ubiquitination involves several enzymes: ubiquitin-activating enzyme (E1), ubiquitin-coupling enzyme (E2), ubiquitin-ligase (E3), and deubiquitination enzyme (DUBs).

Ubiquitin-related enzymes	Effect
E1	E1 activates ubiquitin molecules in an ATP-dependent manner by forming E1-ubiquitin thioesters.
E2	E2 dominates the determination of ubiquitination and polyubiquitination and attaches ubiquitin peptides to the substrate.
E3	E3 determines substrate specificity and covalently binds ubiquitin carried by E2 to the target protein, thereby triggering degradation or modification of the target protein.
DUBs	Reversal of the ubiquitination of the substrate, cutting the ubiquitin chain from the substrate protein into a single reusable ubiquitin fragment.

The stability, functional activity, and interaction of the modified proteins may change, but the modification is a reversible PTM process that can be removed by the UPS (Ubiquitin-proteasome system) or DUBs (Deubiquitinating enzymes) (100). The UPS can remove proteins that have been damaged or are no longer needed in cells (tumor suppressor proteins, cell cycle regulatory proteins, etc.), which is an energy-consuming but highly efficient way to degrade proteins. When the substrate has four or more UB or UBLs, the 26s protease hydrolyzes it into peptide chains, releasing ubiquitin monomers that can be recycled (101). DUBs catalytic deubiquitination modification makes the ubiquitination process maintain the dynamic balance of the cellular process. After DUBs are bound to the ubiquitin substrate protein complex, the ubiquitin chain is broken by severing the isopeptide bond between Lys and the C-terminal of ubiquitin, and the ubiquitin monomer is free and can be collected, thus starting the next round of ubiquitination.

There are approximately over 100 types of DUBs, including ubiquitin-specific proteases (USP), ubiquitin-c-terminal hydrolases (UCH), and so on (102, 103).Different DUBs play different roles in inflammation and cancer development by affecting their substrates’ protein stability, enzyme activity, or subcellular localization (104). The USPs family is the most commonly studied DUB family. USPs regulate protein activation by dissociating single or multiple ubiquitin chains from ubiquitinated substrates (105). In malignant tumors, USP18 is elevated and activates AKT/mTOR signaling, promotes phosphorylated AKT (p-AKT) and p-mTOR protein expression, leading to cancer cell proliferation and migration (106). USP11 controls its stability by promoting deubiquitination of a residual protein (VGLL, a tumor suppressor) and exerts its tumor suppressor effect through the VGLL4/YAP-TEAD regulatory ring (107). Overexpression of USP14 inhibits I-κB and increases NF-κB phosphorylation while increasing cancer cell migration, invasion, and EMT (108). Under selective conditions, USP7, USP10, USP29, USP42, and other DUBs regulate p53 ubiquitination levels (109).

## 5 The relationship between ubiquitination, MSC/MSC-Ex, and IBD

**Figure 3:**
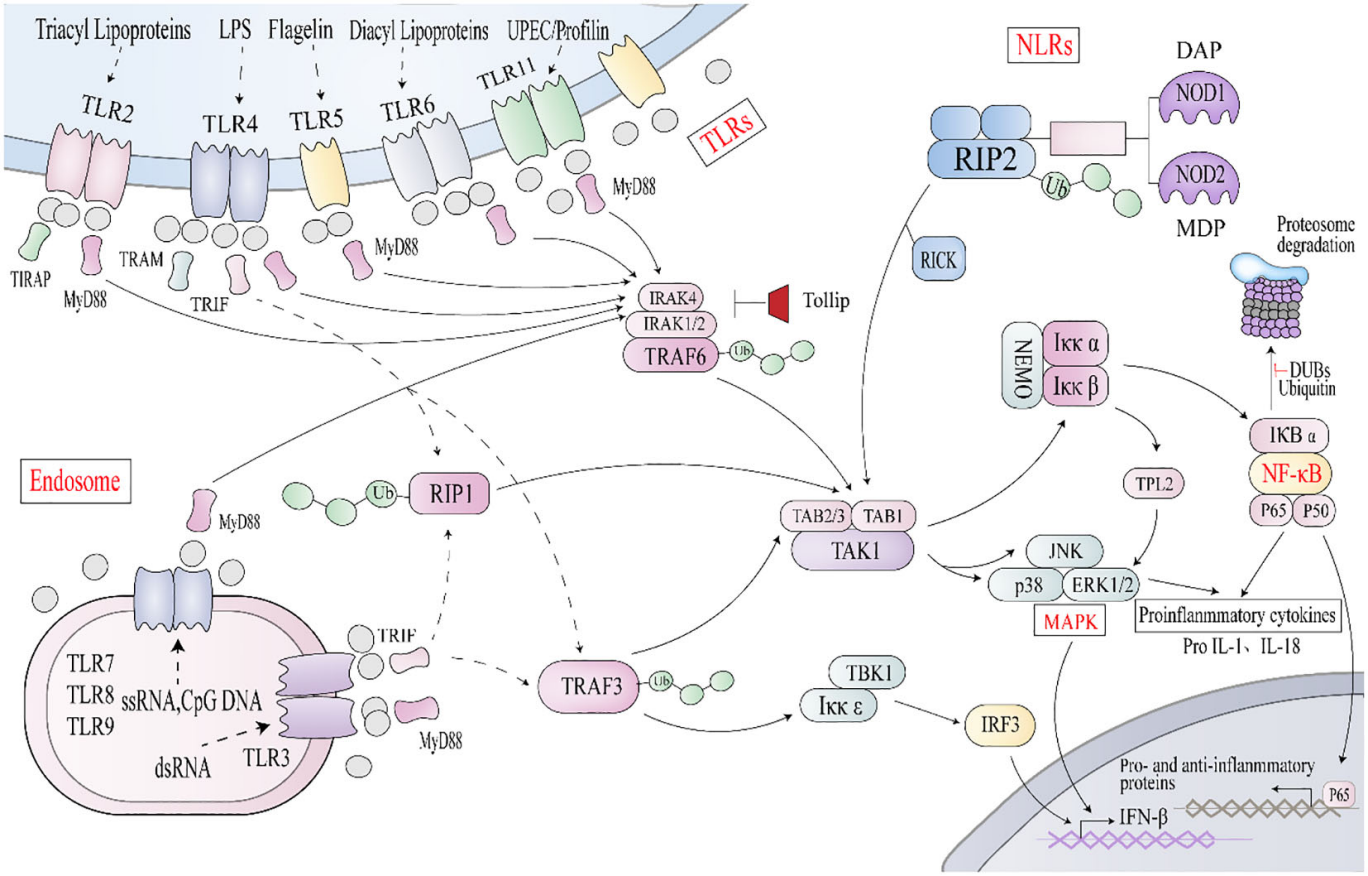
Key signaling pathways associated with IBD ① TLR stimulation triggers MyD88 to interact with IRAK4 (interleukin-1 receptor-associated kinase 4) and ② NLRs interact with RIP2, causing TRAF3, TRAF6, and Lys63 polyubiquitinizes RIP1, to recruit TAK1/TAB2/3 complex or IkK complex. This triggers the activation of NF-κB and mitogen-activated protein kinase (MAPK) signaling pathways, promoting the transcription of proinflammatory and anti-inflammatory genes. Endosomal TLRs transmit signals through a TRIF-dependent pathway, and TRIF, along with RIP1 and TRAF3, activates TAK1 or IKKϵ, leading to phosphorylation of IRF3 and expression of interferon β.

The occurrence of IBD is mainly due to the abnormal amplification of the immune response of the intestinal mucosal immune system to microbial antigens from the gut in some specific populations carrying susceptible genes under certain circumstances, resulting in inflammatory damage of the intestinal mucosa. In 2001, NOD2 was identified as a susceptibility gene for CD due to its polymorphism (110). In addition, approximately 240 gene loci have been found to be associated with IBD susceptibility and occurrence, of which about 30 are CD and UC (111, 112). The loss of balance between proinflammatory and anti-inflammatory factors leads to the activation of NF-κB, TNFα, NLR, and TLR pathways to expand the range of inflammation and promote the development of IBD (**Figure 3**). According to mechanistic studies, a variety of signaling pathways have regulatory effects on IBD, but the specific activation, effects, and regulatory mechanisms largely remain unclear. Therefore, further exploratory studies on the pathogenesis of IBD, new targets for its treatment, and diagnostic biomarkers are needed.

The immune system is mainly composed of the innate and adaptive systems (113, 114). Ubiquitination plays a vital role in the regulation of innate immune signal transduction, but so far, only Lys residues have been identified as ubiquitination sites in innate immune signal molecules. Whether ubiquitination of non-lysine residues plays a role in innate immune signal transduction needs further study. Adaptive immunity relies on specific immune cells (T and B lymphocytes), mediating humoral and cellular immunity, and is characterized by the presence of highly specific antigen recognition receptors, namely T cell receptors (TCR) and B cell receptors (BCR). The regulatory effects of ubiquitination on immune cells are varied and not fully understood. MSCs and MSC-Exs have been shown to target the treatment of inflammatory diseases, including IBD, asthma, and rheumatoid arthritis (115, 116). Ubiquitination is involved in the inflammatory response in IBD, serving as a potential therapeutic target through the modulation of MSC and MSC-Ex.

### 5.1 NF-κB signaling, ubiquitination, and MSC/MSC-Ex

Protein ubiquitination can regulate various signal-mediated inflammatory responses and plays an important role in the occurrence, development, and outcome of inflammatory diseases such as IBD. As a key component of the innate immune response, the NF-κB signaling pathway is one of the ultimate targets for regulating multiple upstream signaling pathways. Research has shown that the NF-κB signaling pathway is crucial in inducing pro-inflammatory gene expression, regulating inflammasome, activating inflammatory T lymphocytes, and innate immune cell differentiation in IBD patients. The activation of the NF-κB signaling pathway requires RIP2 and TAK1-mediated polyubiquitination of NEMO (NF-κB essential regulator kinase, also known as IKγ) and phosphorylation of the kappaB kinase inhibitor (IKK) complex consisting of NEMO, IKKα, and IKKβ. IKKs can be activated by bacterial lipopolysaccharide (LPS), tumor necrosis factor-α (TNF-α), IL-1β, and various physical and chemical stresses (117). Phosphorylated IκB family proteins become targets of K48 polyubiquitination-dependent proteome degradation, releasing active NF-κB molecules into the nucleus. The classical NF-κB pathway is dominated by the action of IKKβ, which phosphorylates IKKβ family members such as IKKα and P105. When stimulated by LPS, IKK promotes nuclear translocation of p65 and p50 and induces expression of inflammatory factors such as TNF-α, IL-1β, IL-6, and IL-12, further leading to tissue damage (118).

Ub plays a crucial role in regulating the activation of NF-κB signaling. Relevant studies have shown that ubiquitin modification can affect intestinal mucosal inflammatory injury and intestinal epithelial cells’ permeability and apoptosis by regulating the NF-κB signaling pathway. TRAF6, one of the E3 ubiquitin ligases, contains a highly conserved RING domain important for activating the NF-κB pathway (119). Chen et al. report that TRAF6 could only activate IKK under the condition of K63 polyubiquitination and that RING domain mutation results in the loss of ubiquitin ligase activity of TRAF6 and the failure to activate IKK (120). NIK is a central kinase in the non-classical NF-κB pathway and is also involved in the classical NF-κB pathway (121). Inhibition of the E3 ligase TRIM16 effectively increases the formation of K48-linked polyubiquitin chains on NIK (122). NEDD4 is associated with chronic inflammatory diseases, and a single rs8032158 transcription variant (TV3) in the NEDD4 genome has been found in keloid patients to activate the NF-κB signaling pathway by binding to the connexin RIP and highly selectively expressed (123, 124).

MSC and MSC-Ex regulate the NF-κB signaling pathway to influence the treatment of IBD. According to the current literature, the degradation of IκB is mainly dependent on neddylation, and when cullin1 activation is blocked, the accumulation of IκB leads to inhibition of NF-κB activity. Wang et al. verified that miR-326 in hucMSC-Ex inhibits the binding of free NEDD8 and substrate protein cullin1, preventing the expression of E1, E2, and E3 enzymes during neddylation formation (125). The hucMSC-Ex also inhibits the activation of the NF-κB signaling pathway, alleviating IBD. In addition, Qi et al. showed that serum-preconditioned adipose-derived MSCs (CM-AcMSC) significantly prevent the phosphorylation of p65 and IκB in colon cells of DSS-induced colitis model in rats, up-regulate the expression of MUC2 and tight-junction proteins such as ZO-1, claudin-1, and occludin, and protect the integrity of colon mucus (126). These results suggest that CM-AcMSC can significantly mitigate inflammation in colitis rats. A similar study showed that bone marrow MSCs (BM-MSCs) down-regulate the expression of NF-κB p65 mRNA in the colonic mucosa, suggesting that BM-MSCs may influence TNBS-induced colitis by regulating NF-κB mediated proinflammatory response (127). DC-derived exosomes activate the NF-κB signaling pathway via exosomal miR-146b to improve intestinal barrier function in DSS-induced colitis (128). Thus, a number of studies indicate that MSC and MSC-Ex play a role in the treatment of IBD by down-regulating NF-κB signaling, but the specific mechanism related to their regulation of NF-κB signaling via ubiquitination needs further study.

### 5.2 TLR signaling, ubiquitination, and MSC/MSC-Ex

TLRs are pattern recognition receptors. As an important part of the innate immune system, TLRs activate a series of downstream signals after recognizing pathogen-associated molecular patterns (PAMPs), inducing the secretion of inflammatory cytokines, chemokines, and type I interferons (129, 130). After binding to different stimuli, most TLRs initiate signal transduction by recruiting the adaptor protein MyD88, which contains the TIR domain and is a common important adaptor protein of most TLRs and a variety of envelope receptor-mediated signaling pathways. It plays a role in recruiting downstream kinases and regulating signal transmission; ① In the MyD88-dependent pathway, MyD88 recruits IL-1 receptor-associated kinase-4 (IRAK4) to attract TLR, and the MyD88-IRAK4 complex recruits IRAK4 substrate IRAK2 or related IRAK1 to realize Myddosome (131, 132). This protein interacts with TRAF6, self-ubiquitination modification of TRAF6, activates TAK1, and ultimately stimulates NF-κB and JNK/P38/ERK signaling pathways, which participate in the colon inflammation in IBD; ② MyD88 signaling pathway can also be used as another pathway for TLR to induce inflammation. After TLR activation, it can activate the toll-like receptor-associated activator of interferon (TRIF) and TRAF3, resulting in NF-κB inhibiting the recruitment of protein kinase ϵ/tank-binding kinase 1 (IKϵ/TBK1), inducing the phosphorylation of IRF3 and the expression of interferon-β, and playing an antiviral role. TRIF- and MyD88-mediated signaling involve a series of ubiquitination events. TRAF3 and TRAF6, as members of the E3 ubiquitin ligase family, play an essential regulatory role in MyD88-dependent and TRIF- (non-MyD88) dependent signal transduction, which can be either a “positive signal” or a “negative signal”. Moreover, TRAF3 and TRAF6 can regulate the activation of inflammation-related signaling pathways through ubiquitin modification effects and initiate the gene transcription of many proinflammatory cytokines, such as IL-1, IL-6, TNF-α, and other transcription factors to activate a variety of immune responses, thereby playing a role in immune defense or relieving inflammation. Nrdp1 directly binds to and polyubiquitinates MyD88 and TBK1 while promoting TLR-triggered macrophages to inhibit the production of proinflammatory cytokines. In addition, Nrdp1 and Smurfp1/2 can catalyze the ubiquitination modification of MyD88 or remove the ubiquitin chain for negative regulation (133, 134). A20 and SIGIRR can also adjust the duration and/or strength of the TLR signal (135–137).

Macrophages are considered classic cells in TLR studies (138). They are the most critical cells in inducing colon inflammation, releasing large amounts of DAMP in damaged intestinal epithelial cells and activating NF-κB in intestinal macrophages signaling pathways that promote the secretion of inflammatory factors such as TNF-α and IL-1β, lymphocytes and monocytes, recruit chemokines (CCL-17 and CCL-24) and NO, and promote colon damage. Duan et al. demonstrated that LIM domain 7 (LMO7) is an important molecule that regulates macrophage polarization and inhibits intestinal inflammation in a DSS-induced IBD model (139). When proinflammatory activates macrophages, PFKFB3(6-phosphofructose-2-kinase/fructose-2,6bisphosphatase 3) promotes glycolysis by increasing the activity of phosphofructokinase-1 (PFK1). LMO7 promotes the degradation of PFKFB3 through K48-related ubiquitination, thereby effectively preventing excessive inflammatory response of macrophages and protecting tissues from inflammatory damage.

In the regulation of IBD by MSC and MSC-Ex, Liotta et al. demonstrated that the binding of TLR3 or TLR4 to MSCs can modulate their immunosuppressive activity against T lymphocyte proliferation, thereby restoring an effective T cell response during infections (140). Studies have shown that MSCs can inhibit the LPS/TLR4 signaling pathway, thereby reducing the release of inflammatory factors, improving intestinal symptoms of IBD, and reducing parenteral complications (141). Liu’s team reports that metallothionein-2 in MSC-Exs, a key negative regulator of macrophage inflammatory response, plays an anti-inflammatory role in conjunction with other components of MSC-Exs to maintain intestinal barrier integrity and reduce experimental colitis in mice (142). Other studies indicate that MSC-Ev inhibits the activation of proinflammatory M1 macrophages and promotes their polarization to M2 macrophages, alleviating the inflammatory response and DSS-induced IBD (143). Deng et al. developed a technique that can sustainably release MSC-Exs for regenerative purposes using an *in situ* synthetic biotin-modified MSC-Ex (Bio-Ex) self-assembled biotinylation (144). The Bio-Ex can be taken up by macrophages and play an immunomodulatory role similar to MSC-Ex, promoting the polarization of macrophages to the M2 phenotype. Perhaps ubiquitin is involved in alleviating IBD through the TLR signaling pathway, or the regulation of macrophage inflammatory response by MSCs and MSC-Exs are linked with TLR/ubiquitination. However, the direct link to this hypothesis remains to be proven.

### 5.3 NLR signaling, ubiquitination, and MSC/MSC-Ex

Nucleotide-binding and oligomeric domain (NOD)-like receptors (NLRs) are a type of PRRs that are primarily distributed in the cytoplasm and have four broad classes of functions: inflammasome assembly, signal transduction, transcriptional activation, and autophagy (145–147). Cumulative data suggest that NLRs play a vital role in a variety of autoimmune diseases, such as IBD, multiple sclerosis (MS), and systemic lupus erythematosus (SLE) (148).

The C-terminal of NLRs (except NLRP10) contains a leucine-rich repeat sequence (LRR) that specifically recognizes the PAMPs’ or DAMPs’ molecular pattern. Based on the unique functional features of the N-terminal effector domain, NLRs can be divided into five subfamilies: NLRA, NLRB, NLRC, NLRP, and NLRX1. The structure of the NLRA subfamily includes CIITA (class II transcription activator), the activation of which is dynamically regulated by a series of post-translational modifications, such as acetylation, phosphorylation and ubiquitination.

Ubiquitination is involved in activating and terminating NOD signaling cascades. After NOD1 and/or NOD2 activation, the oligomerization of the NACHT domain between the NLRs N-terminal and the LRR domain activates and recruits the interacting proteins to form a semallome including RIP2(also known as RICK). E3 ubiquitin ligase cIAP1 forms a ubiquitin chain with RIP2, catalyzes the ubiquitination of RIP2, induces the activation of the TAK1 complex, triggers the activation of NF-κB and MAPK signaling pathway, and promotes the transcription of proinflammatory genes (IL1β, IL-18). Studies have shown that when the NOD1-RIP2 signaling pathway is activated, hybrid ubiquitin chains containing M1-Ub and K63-Ub bonds are rapidly produced, and hybrid ubiquitin chains may affect the deubiquitination rate of K63-Ub and M1-Ub chains. This affects the duration of the innate immune response (149). After NOD2-RIP2 activation, it promotes K)63-linked polyubiquitination of NEMO, thereby promoting the recruitment of TAK1 and activating the NF-κB signaling pathway (150, 151). TRAF4 is an E3 ligase that has been shown to negatively regulate NOD2 signaling (152). Moreover, autophagy-associated protein 16-like-1 (ATG16L1) negatively regulates NOD-driven inflammatory responses by interfering with RIP2 junction polyubiquitination (153).

In addition to NOD-mediated activation of NF-κB and MAPK, inflammatory bodies (NLRs) can also regulate inflammatory responses through ubiquitination modification (154). The NLRP3 inflammasome (NOD-, LRR- and pyrin domain protein 3) is the most studied, an intracellular polymeric protein signaling complex that participates in the innate immune system and plays an important role in maintaining intestinal homeostasis and preventing colitis (155, 156). NLR proteins such as NLRP3 detect pathogens or danger signals and trigger the assembly of caspase-1 inflammasome, leading to the processing and secretion of IL-1β and IL-18, promoting the proliferation and differentiation of pro-inflammatory macrophages and tissue damage. It also contributes to pyrosis, either directly or through ASC junction proteins. A study found that in the GVHD model, activated NLRP3 inflammasome stimulates choline metabolized TMAO (trimethylamine N-oxide) to induce M1 macrophage polarization, leading to the differentiation of T1 and T17, which aggravated the disease (157). Ubiquitination also plays a vital role in the NLR signaling pathway. According to Xu et al. found that E3 ubiquitin ligase gp78 mediates the mixed ubiquitination of NLRP3 and inhibits the activity of NLRP3 by preventing the oligomerization and subcellular translocation of the NACHT domain of NLRP3, thereby reducing the activation of inflammasome and harmful effects (158). Mai et al. confirmed that promoting mitochondrial autophagy driven by E3 (Parkin) and inhibiting the activation of colonic NLRP3 inflammasome has an inhibitory effect on mouse DSS-induced colitis (159). The E3 ligase TRIM31 binds NLRP3 directly and promotes K48-linked NLRP3 ubiquitination and proteasome degradation, maintaining low NLRP3 expression and preventing unwanted inflammasome activation (160). Moreover, interference with A20 inhibits macrophage proliferation and M2-like polarization by activating the NLRP3 inflammasome pathway (161).

Studies report that MSC-Ex miR-378a-5p targets and blocks NLRP3 inflammasome activation in macrophages, leading to Caspase-1 cleavage and IL-1β and IL-18 reduction, delaying pyroptosis cell death and improving IBD (162). MSC-Ex alleviates colitis by increasing FXR in the colon, which binds to the NLRP3 inflammasome and inhibits the activation of inflammasome components (163, 164). The regulation of IBD inflammation by ubiquitination and the treatment of IBD with MSCs and MSC-Exs also involve NOD and NLR signaling pathways, and there may be some unknown relationships that require further studies.

### 5.4 T cell activation, ubiquitination, and MSC/MSC-Ex

When microbes and metabolites interact with pattern recognition receptors (PRR), such as pregnane X receptor (PXR) and TLR, there is activation of signaling pathways and key proteins that control mucosal barrier and intestinal immune functions. When pathogens invade the human body, TLRs guide mucosin-2 (MUC2) in the intestinal mucus layer to prevent intestinal pathogens and their secretions from penetrating the mucosa, thus playing an essential role in preventing inflammation (165–167). During the progression of IBD, activated T lymphocytes infiltrate the inflamed site and produce a variety of cytokines, further aggravating intestinal inflammation. T cells in IBD patients are composed of pro-inflammatory effector subsets (Th1, Th2, and Th17) and/or regulatory T (Treg) cells that have immunosuppressive effects and maintain intestinal homeostasis (168, 169). The proportion of CD8^+^ T suppressor cells and CD4^+^ T helper cells in the lamina propria and epithelium of the intestinal tract of IBD patients is usually normal, but the activated cells showed an increasing trend. The cytokine IL-13 secreted by Th2 cells plays a role in UC, and Th17 is involved in the pathogenesis of IBD through the production of IL-17A. On the other hand, Treg cells suppress the immune response and prevent self-hyperimmunity, and IL-10 produced by Treg cells inhibits the proinflammatory cell Th17 in the gut. In contrast, the elimination of IL-10 receptors in Treg cells leads to Th17 dysregulation and colitis (170).

Both programmed death protein 1 (PD-1) and cytotoxic T lymphocyte-associated antigen 4 (CTLA-4) appear to be key markers for controlling T cell tolerance and have negative regulatory effects on T cell immune function (171, 172). CTLA-4 is mainly involved in the early stages of T cell immune responses in lymph nodes (173, 174), while PD-1 is mainly involved in the late stages of T cell immune responses in peripheral tissues. PD-1 is expressed by activated T cells and downregulates T cell effector function after binding to its ligands PD-L1 and PD-L2 on antigen-presenting cells (175). Intestinal epithelial cells of IBD patients overexpress PD-L1 and PD-L2 (176), and PD-1 blocking can reverse the *in vitro* inhibition of effector T cells mediated by Treg. Most studies have shown that Tregs constitutively express CTLA-4, which is considered important for its inhibitory function (177).In a FoxP3^+^ conditional knockout mouse model with CTLA-4 deletion, Wing et al. clearly demonstrated that CTLA-4 deficiency in FoxP3^+^ Treg cells impairs its inhibitory function (178). Experimental data of Takahashi et al. showed that the signals sent by TCR and CTLA-4 may activate CD25^+^CD4^+^ regulatory T cells, thereby transmitting negative signals of activation and proliferation to other T cells. These possibilities are currently being investigated (179).Therefore, maintaining a balanced ratio of Th17 and Treg cell populations is essential for maintaining the intestinal immune system (180).

There is growing evidence that ubiquitination-degrading proteins play a role in suppressing immune responses by targeting the destruction of signaling proteins and pro-transcription factors. Dysfunction of E3s ubiquitin ligase, the final step in catalyzing ubiquitin attachment to substrate proteins, can lead to abnormal T cell activation and loss of tolerance to autoantigens, resulting in immune dysfunction (181), and mice lacking these proteins show significant inflammation and/or autoimmune-like symptoms. Several E3 ubiquitin ligases, most notably Itch, Roquin, and CBI-b, have been shown to regulate T cell activation. Ramon et al. found that NEDD4 family interacting protein 1(Ndfip1) is a regulatory protein of Itch, and the combination of the two can make JunB ubiquitination (182). JunB is a transcription factor that promotes the expression of Th2 cytokines IL-4 and IL-5, Therefore, blocking the production of IL-4 and IL-5 may be one of the targeted mechanisms of Th-cell associated gastroenteritis and IBD. In addition, deubiquitinating enzymes USP22 and UCHL1 have been shown to deubiquitinate and stabilize PD-L1 protein (183, 184). Proteolytic targeting chimeras (PROTACs) can recruit the E3 ligase RNF43 to the lysosomes that induce PD-L1, promoting its ubiquitination and subsequent degradation (185). Fujiwara et al. first demonstrated that Cbl-b deficiency in mice leads to functional resistance of T cells and NK cells to PD-L1/PD-1-mediated immune regulation and mild autoimmune reactions (186). To date, there has been no report of any E3 directly ubiquitinating CTLA-4 or CTLA-4 ubiquitination sites. However, multiple studies have shown a strong correlation between CTLA-4 ligation and the function and expression of Cbl-b (187). It is currently known that the main CTLA-4 inhibitory function is mediated by key T cell inhibitory E3 ligases, namely Cbl-b, Itch, and GRAIL (188). Overall, Cbl-b not only mediates CTLA-4 signaling but also mediates PD-1-induced immunosuppression.

Exosome-mediated immune response has been shown in the pathophysiology of IBD (189). The effect of MSCs on T cells seems to depend on the MSC/T cell ratio: a high MSC/T cell ratio has a strong inhibitory effect, while a low ratio may enhance T cell proliferation. It is known that T-box (T-BET) and retinol-associated orphan receptor γ(t) in T cells (RORγT), as central regulators of Th1 and Th17 cells, respectively, are pathogenic factors for IBD progression, while MSCs may down-regulate Th1-Th17driven autoimmune and inflammatory responses by influencing the expression of T-bet and RORγt (190). HucMSC prevents experimental colitis by increasing the number of CD5^+^ B cells and CD5^+^Bregs that produce IL-10, and restores Treg/Th17/Th1 imbalances (191). Olfactory Ecto-Mesenchymal Stem Cell-Derived Exosomes (OE-MSC-Exs) regulate T cell responses and have a significant inhibitory function on CD4^+^ T cells, presenting a novel cell-free therapy for IBD and other inflammatory diseases (192).

### 5.5 TGF-β signaling, ubiquitination, and MSC/MSC-Ex

Impairment of the TGF-β signaling pathway is associated with the development of intestinal inflammation in experimental models and IBD patients. TGF-β is an immunosuppressive cytokine produced by various cell types (immune cells, nonhematopoietic cells) and activated by integrins. There are three types of TGF-β in mammals: TGF-β1, TGF-β2, and TGF-β3. TGF-β receptors are widely expressed in body tissues and cells; therefore, members of the TGF-β family regulate the proliferation, differentiation, apoptosis, and inflammation of many cells. TGF-β signaling requires two transmembrane receptors, RI and RII types, that have serine/threonine kinase activity. When the active TGF-β ligand binds to the RII receptor, it activates the kinase activity of the RI receptor and the recruitment of Smad protein, inducing Smad complex formation, nuclear transport, and Smad DNA binding (193). Smad works with universal transcription factors (GTF), -determining transcription factors (LDTF), and other driving factors or helper proteins to regulate target gene transcription.

The TGF-β signaling pathway has been studied for its effective regulatory and inflammatory activity (194). In intestinal immunity, TGF-β inhibits intestinal bacterial antigens’ inflammatory response and helps induce immune tolerance. IBD is characterized by abnormal TGF- β signaling (195). High expression of Smad7 in CD4^+^ T cells is associated with severe colitis (196). Studies have shown that sCYLD (a short splicing form of CYLD) mediates ubiquitination of K63 junctions and nuclear translocation of Smad7 and that the sCYLD-Smad7 complex inhibits TGF-βsignaling in CD4^+^ T cells (197). It is worth noting that Smurf1 and Smurf2, members of the E3 ubiquitase family, are critical negative regulators of the TGF-β signaling pathway (198). Smad7 recruits E3 ubiquitin ligases such as Smurf1, Smurf2, and NEDD4L to TGF-β receptors, promoting their ubiquitin-mediated degradation. RNF11 may mediate AMSH ubiquitination through the formation of the Smurf2/RNF11 complex, leading to its degradation by the 26S proteasome, negatively regulating TGF-β signaling (199).

TGF-β1 in exosomes is thought to have therapeutic potential in IBD. Exosomes produced by TGFβ1 gene-modified DCs can inhibit the development of IBD by inhibiting Th17 (200). Another study showed that TGF-β1-modified exosomes (TGF-β1-Exs) induce CD4^+^ Foxp3^+^ Tregs, reducing the proportion of Th17 in lymphocytes at the site of inflammation, mitigating the inflammatory response in a mouse model of colitis (201). In experimental models and IBD patients, impaired TGF-β signaling pathways have been associated with the development of intestinal inflammation. OE-MSC-Exs can regulate cell proliferation, decrease the levels of inflammatory cytokines IL-17 and IFN-γ, and increase the inhibitory cytokines TGF-β and IL-10, suggesting that OE-MSC-Exs may effectively alleviate the severity of experimental colitis by inhibiting effector T cells and enhancing regulatory T cells (192). Ma ZJ et al. found that MSC-Ex decreased the concentrations of IFN-γ, TNF-α, and IL-1β and increased the secretion of TGF-β1 and IL-10 by up-regulating anti-inflammatory response and down-regulating inflammatory response (202). It is suggested that MSC-Ex shows therapeutic power in a mouse model of DSS-induced colitis by inhibiting inflammatory mechanisms. Pd-MSC-Ev inhibits TGF-β1-induced inflammatory cytokine secretion and fibrotic marker expression, suggesting that MSC-EVs is expected to be a promising anti-fibrotic drug (203).

### 5.6 Deubiquitase and E3 ubiquitin ligase in IBD and MSC/MSC-Ex modulation

A variety of deubiquitases and E3 ubiquitin ligases are involved in regulating the process of IBD. USP15 binds to Lys48-linked ubiquitin chains to achieve deubiquitination, inhibiting the degradation of TAB2 and TAB3, thus hindering the selective autophagy degradation mediated by autophagy cargo receptor 1 (204). The proteasomal-associated deubiquitinase USP14 is involved in the negative regulation of the type 1 IFN signaling pathway and can also inhibit the activation of the NF-κB signaling pathway by deubiquitinating K63-linked retinoid-inducing gene I (RIG-I) (205, 206). USP19 negatively regulates the activation of TAK1-TAB1 dependent NF-κB signaling pathway by specifically removing TAK1 coupled Lys63 and Lys27 linked polyubiquitin chains, resulting in impaired TAK1 activity and destruction of the TAK1-TAB2/3 complex (207). The E3 ubiquitin ligase TRIM56 induced by type I IFN interacts with STING and targets STING for K63 junction polyubiquitination, recruitment of TBK1, and induction of IFN-β to induce innate immune response (208).TRIM32 can also interact with STING on mitochondria and ER, facilitating STING interaction with TBK1 (9). Upregulation of surface MHC Class II (MHCII) and co-stimulatory molecules such as CD80 and CD86 leads to DC maturation, E3 ligase membrane-associated RING-CH-1 (MARCH1) promotes endocytosis and lysosomal degradation of MHCII and CD86 by ubiquitinating them. Thus limiting the antigen-presenting capacity of dendritic cells (209–211).

Sun et al. showed that USP11 plays a catalytic and non-catalytic role in regulating the stability of IκBα, thereby negatively regulating TNF-α induced NF-κB activation (212). Deubiquitination of USP26 has been found to stabilize SMAD7, leading to reduced TGF-β signaling (213). Moreover, RNF182 promotes the degradation of cytoplasmic p65 through K48 ubiquitination, inhibiting inflammatory responses (214). In patients with IBD, up-regulated USP16 expression levels can be found in macrophages. When stimulated by LPS or TNF-α, USP16 specifically removes Lys33-linked IKKs polyubiquitin chains and promotes IKK-β-mediated phosphorylation of P105, leading to autoimmune responses and the development of IBD (215). Li Y et al. confirmed that CVMSC-Exs promote trophoblast migration and proliferation by up-regulating TRIM72 expression, thereby promoting P53 ubiquitination, proteasome degradation, and reducing cell apoptosis (216). Patients with inflammatory bowel disease (IBD) have higher levels of angiotensin-converting enzyme 2 (ACE2) expression in the gut (217).ACE2 deubiquitination mediated by deubiquitination enzyme UCHL1 and ACE2 SUMO mediated by E3 SUMO protein ligase PIAS4 can increase ACE2 protein levels, while AP2-mediated lysosomal degradation can decrease ACE2 protein levels (218, 219). NEDD4 has been shown to be involved in Ev biosynthesis (exosome production), and NEDD4 is a novel GSDMD interacting protein. Bulek et al. ‘s data suggest that GSDMD uses selective autophagy components (including LC3+ vesicles) to mediate NEDD4-dependent sEV biosynthesis for IL-1β output, thereby supporting the interaction between autophagy and exosome biosynthesis (220), Regardless of the proposed mechanism, exosomes can alleviate DSS-induced colitis in mice by controlling ubiquitin modification levels (17). However, at present, there is little literature on the clear association between deubiquitin/E3 ubiquitin ligase and MSC/MSC-EX in IBD disease, and the specific mechanism still needs to be further explored (17, 221–223).

## 6 Conclusion

Scientists have been exploring more effective and easier ways to treat IBD, mainly focusing on the interaction between genetic, environmental, immunological, and gut microbial factors, to discover further fundamental mechanisms of IBD occurrence and preventive strategies. The aim is to reduce patients’ medical and disease burden and improve the quality of life of affected individuals (224). Despite the best efforts, the application of MSCs and MSC-Exs remains a black box filled with unknown secrets. Studies have reported that treatment with MSCs or similar cells may promote the likelihood of cancer in patients; although this risk is unlikely to exist, there is still a chance of occurrence (225). As research continues to deepen and clinical trials advance, we hope to better understand the risks and potential benefits of exosome treatment for patients. Thus, the search for new, safe, efficient, and low-cost treatments for IBD, including MSC-Exs, is still underway.

Several experimental conclusions have proved that ubiquitin modification is involved in regulating important signaling pathways, such as NF-κB, NOD, TGF-β, and TNF-α. In addition, the dysregulation of components of the ubiquitination system often leads to various diseases such as cancer, IBD, and other autoimmune diseases. Some ubiquitin enzymes are known to directly regulate various inflammation-related transcription factors from the Smad, p53, Jun, and other families, and the ubiquitination-mediated degradation of signaling intermediates is an essential means to terminate inflammatory responses (226). However, how ubiquitination mediates the transmission and function of inflammatory signals to trigger the occurrence of IBD is largely unexplored and requires further studies. Moreover, the link between ubiquitination, IBD, and MSCs/MSC-Exs could provide an experimental basis for a novel therapeutic target and subsequent clinical application. More exploratory studies are needed in this area.
